# Microbiota in interstitial cystitis/bladder pain syndrome: evidence and opportunities

**DOI:** 10.1172/JCI197858

**Published:** 2025-09-02

**Authors:** David J. Klumpp

**Affiliations:** Departments of Urology and Microbiology-Immunology, Feinberg School of Medicine, Northwestern University, Chicago, Illinois, USA.

Microbiota are essential for normal biologic function and play central roles in diverse pathologies. They also modulate pain responses and are increasingly understood to reside in niches throughout the genitourinary (GU) tract, including as urinary microbiota. This, in turn, suggests that microbiota may drive or modulate symptoms of urologic chronic pelvic pain syndrome (UCPPS). UCPPS is a chronic visceral condition that includes interstitial cystitis/bladder pain syndrome (IC/BPS, or IC), which primarily affects women, and chronic prostatitis/chronic pelvic pain syndrome (CPPS) in men (1).

UCPPS is marked by chronic pelvic pain and comorbid anxiety and depression, yet no clinically useful biomarkers or effective therapies exist (1). Many patients with IC also experience significant voiding dysfunction, with increased urinary frequency and urgency. Bladder inflammation is variably associated with IC and IC symptoms, most notably partially activated mast cells residing under the urothelium that lines the bladder and in proximity to sensory nerve fibers (2). Patients with IC are often subclassified by findings on cystoscopic evaluation, including Hunner’s ulcers in a subset of patients, and recent studies of clonal B cell populations within Hunner’s lesions raise the specter of autoimmune dysfunction among some patient groups (3). Moreover, the flagship National Institute of Diabetes and Digestive and Kidney (NIDDK) Multidisciplinary Approach to the Study of Chronic Pelvic Pain (MAPP) has revealed key UCPPS patient subgroups based on whether chronic pain localizes to the pelvic region or is a component of widespread chronic pain (1). How these patient subgroups may be affected directly or indirectly by microbiota is still unclear.

## Genitourinary microbiota in IC

The pioneering studies of Brubaker and Wolf using 16S sequencing and extended urine culture techniques dispelled the notion of the bladder as a sterile environment and identified a urinary microbiome (4). Given that bladder sensory pathways are capable of responding to bacteria (5), the presence of a “urobiome” in direct contact with urothelium has elicited many hypotheses regarding urologic health and disease, particularly for conditions of bladder dysfunction. However, the role of the urobiome in IC is yet unclear. Although two studies reported an association, several failed to associate the urobiome with IC, including MAPP (6, 7). Likewise, the vaginal microbiome has not been associated with IC, so connections between IC/BPS and GU microbiota remain elusive.

## Gut microbiota in IC

Several studies now support involvement of gut microbiota, in contrast to GU microbiota, in UCPPS. In 16S sequencing data obtained from fecal samples of 17 healthy female control individuals and 17 female patients with IC, extended random forest was used to identify taxa with differential abundance that associated with symptom severity as quantified by the female-specific MAPP GU pain index questionnaire (8). Twenty-six taxa exceeded a relevance threshold of 70% distributed across diverse phyla. Several taxa were subsequently validated using species-specific primers and described as “deficient in IC pelvic pain” (DIPP), constituting an interacting network that included *Eriocheir* *sinensis*, *Odoribacter* *splanchnicus*, *Faecalibacterium* *prausnitzii*, and *Collinsella* *aerofaciens*. Metabolomics and subsequent pathway analyses identified altered lipid metabolism, including deficiency in biosynthesis of the short-chain fatty acid (SCFA) butyrate, consistent with *O*. *splanchnicus* and *F*. *prausnitzii* being producers of SCFAs that are also implicated in gut health. Supporting a role for dysbiosis in UCPPS generally, studies in men revealed 15 taxa with differential abundance between control and CPPS samples, with patients showing marked deficiency in *Prevotella,* a genus associated with SCFA production (9).

Recent studies utilized Mendelian randomization to correlate taxa with IC-associated SNPs (10, 11), and *Butyricimonas* and *Coprococcus* were identified. Like *F*. *prausnitzii,* *Butyricimonas* and *Coprococcus* are producers of SCFAs that are also associated with gut health. Thus, in studies of the gut microbiome in IC, there is an emerging picture of deficiency among patients with IC/BPS for microbes that produce SCFAs and promote gut health.

Clinically relevant models bolster the potential role for gut microbiota in UCPPS. Pain is technically a patient-reported experience, so bladder-associated pelvic pain in mice is quantified most commonly by observing rodent behavioral correlates of pain, such as visceromotor response (VMR) to bladder distension of increased abdominal muscle electromyographic activity under mild anesthesia or referred mechanical allodynia manifested as enhanced responses (e.g., jumping, grooming, withdrawal) to tactile stimulation of the pelvic region with von Frey filaments. Mice lacking acyloxyacyl hydrolase (AOAH) mimic many key aspects of IC, including chronic pelvic pain associated with increased VMR and mechanical allodynia of the pelvic region, anxious/depressive behaviors, elevated bladder mast cell levels, and compromised urothelium (12). AOAH-deficient mice exhibit gut dysbiosis, including multiple fermenter taxa and functional deficits in gut transepithelial electrical resistance indicative of “leaky gut,” and fecal metabolomics indicated reduced SCFA levels in AOAH-deficient stool (13). Importantly, transfer of WT stool to AOAH-deficient mice reduced pelvic pain, and AOAH-deficient pain was exacerbated by transfer of anaerobic culture of IC patient stool. Gavage of WT stool also reduced anxious behaviors of AOAH mice. Together, these results demonstrate that manipulation of gut microbiota can rectify key IC symptoms in mice and that IC taxa can modulate pelvic pain. Similar studies in CPPS models suggest that gut microbiota modulation of symptoms may be generalizable across UCPPS.

## Bladder-gut-brain axis in IC

**Figure 1:**
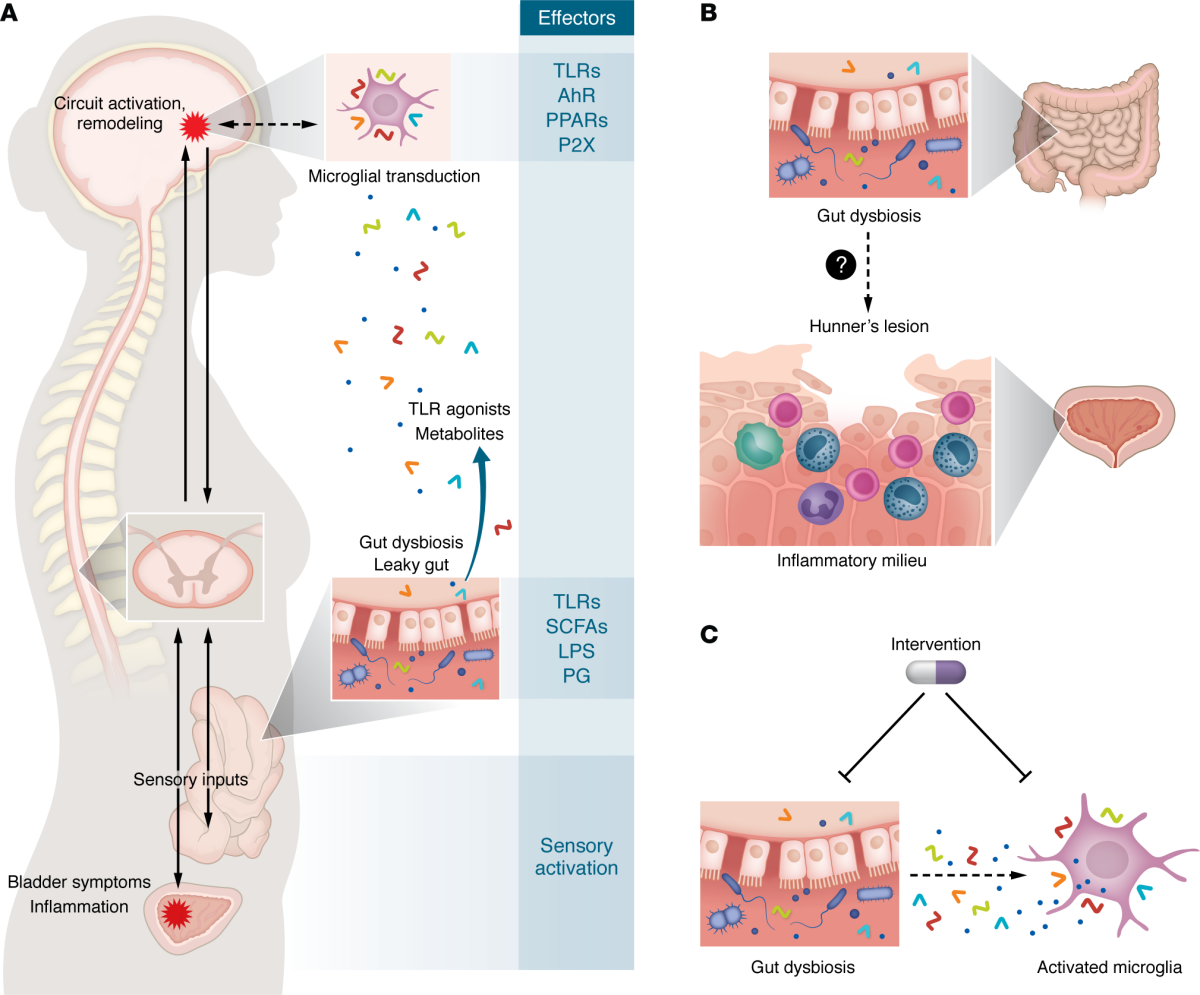
Gut microbiota’s influence on IC symptoms and clinical importance. (**A**) Visceral convergence of bladder and gut sensory pathways mediate organ crosstalk and the bladder-gut-brain axis. Gut dysbiosis in IC may directly influence sensory pathways, or microbial constituents (LPS and peptidoglycan [PG]) and metabolites in circulation can influence pain circuits centrally via microglia. (**B**) Dysbiosis-associated immune dysregulation may impact IC pathology by altering the inflammatory milieu and thus affect bladder mast cells and/or Hunner’s lesions. (**C**) Microglia are potential therapeutic targets for IC by use of drugs that act directly or indirectly.

Bowel symptoms are a common comorbidity among IC patients, and IC symptoms are often exacerbated by comestibles, especially those high in acid or containing caffeine (14, 15). These clinical observations are consistent with early physiologic studies in cats showing that some one-third of dorsal horn neurons receive inputs both from the bladder and gut, and this visceral convergence has been mapped from pelvic viscera to the brain stem (16). Moreover, organ crosstalk between the gut and bladder in rodent models has been demonstrated at the levels of inflammation and pelvic pain, with noxious gut stimuli eliciting and amplifying bladder responses (17). Hence, the gut has long been proposed to play a role in modulating IC symptoms, and gut microbiota are poised to impact IC symptoms along the bladder-brain axis (Figure 1A).

The gut is rich in sensory mechanisms, so microbiota can evoke or modulate gut sensory outputs that impinge upon bladder sensation. For example, neuropod cells of gut epithelium couple directly to neurons, and these gut epithelial sensors mediate responses to foods and microbiota alike (18). Indeed, neuropods express Toll-like receptors as well as receptors for SCFAs, so neuropods can convey signals induced by both taxa of microbiota and products of microbial metabolism. Gut sensation thus has the potential to play an important role in IC symptoms driven chronically by dysbiosis and acutely in response to comestibles.

## Microglia as transducers of dysbiosis in IC

Constituting some 10% of cells in the CNS, microglia are macrophage-like innate immune cells that play critical roles in health and disease and are important in pain responses and pain resolution (19). Microglia respond to environmental cues through a variety of receptors relevant to microbiota with the potential to modulate neuroinflammatory processes. Toll-like receptors mediate microglial responses to bacterial constituents including LPS and peptidoglycan; free fatty acid receptors can respond to SCFAs; and aryl hydrocarbon (AhR) and PPARγ can also mediate microglial responses to SCFAs. Indeed, in the AOAH model of IC, transient pharmacologic ablation of microglia attenuated pelvic allodynia responses, demonstrating a link between microglia and pelvic pain (20). These findings dovetail with imaging studies from patients with chronic pain. PET/MRI brain imaging with tracers specific for the neuroinflammatory marker 18 kDa translocator protein (TSPO) expressed in microglia have implicated microglial activation, as increased TSPO has been observed in multiple pain conditions, including chronic low back pain and fibromyalgia (21). Although TSPO imaging has not yet been performed in patients with IC, together these studies suggest microglia as transducers of dysbiosis involved in chronic pelvic pain and thus potential targets for IC therapies.

## Clinical questions and translational opportunities

With one or more microbiota implicated in IC and impinging upon bladder sensation, clinical questions and therapeutic opportunities arise. Modulation of immunity and pain by gut microbiota is well established (22). As a result, altered systemic and/or tissue-specific immune modulation wrought by dysbiosis may initiate or exacerbate bladder inflammatory processes contributing to pelvic pain. Thus, it will be important to determine how such potential mechanisms of dysbiosis-driven immune dysfunction impact specific IC patient subgroups, especially those diagnosed with Hunner’s lesions or with increased bladder mast cell levels (Figure 1B).

IC remains a clinical entity without effective biomarkers for diagnosis or monitoring of response to therapy, but microbiota provide an opportunity to develop novel IC biomarkers. Stool is a potential source of clinically useful biomarkers for GI conditions, which raises the possibility that it may similarly provide novel UCPPS biomarkers. Indeed, receiver operating characteristic (ROC) curve analyses of IC/BPS indicated multiple DIPP taxa and metabolites with sensitivity and specificity appropriate for consideration as stool-based IC biomarkers (8). To maximize clinical utility and better tailor therapies, further development of DIPP taxa and metabolites requires more detailed studies to identify stool-based biomarkers or combinations thereof that discriminate subtypes of patients with IC.

Gut dysbiosis offers multiple opportunities for therapeutic targeting. However, strict dietary management to mitigate IC symptoms with the “IC diet” (avoidance of acidic, spicy, or other foods that trigger bladder symptoms) may limit the ability to remodel the gut microbiome through diet alone. Fecal microbiota transfer (FMT) with healthy stool has been employed to treat dysbiosis in gut inflammatory conditions, where key aspects of clinical success include achieving ecological success with the establishment of keystone taxa (23). Likewise, given findings of dysbiosis in UCPPS and symptomatic improvement by FMT in clinically relevant IC models, targeting dysbiosis represents a potential therapeutic option in IC, consistent with the amelioration of dysbiosis and thus disruption of its role in visceral pain. Probiotics offer similar potential to rectify dysbiosis by functionally complementing specific deficits such as SCFA production that may benefit patients with IC.

Finally, small-molecule drug therapy targeting neuroinflammatory mechanisms may offer special utility for treating dysbiosis-associated chronic pain (Figure 1C). For example, preclinical studies have shown that the antibiotic minocycline inhibits microglia and reduces pain in animal models, supporting clinical use of minocycline for pain by targeting microglia directly. Initial clinical trials of minocycline for low back pain have shown mixed results, although confounding factors support further studies that optimize participant groups and dosing regimens (24). However, minocycline therapy has the obvious secondary effects of remodeling microbiota. In a rat model of anxiety, minocycline reduced the depressive phenotype, microglia, and proinflammatory cytokines in plasma, while increasing plasma hydroxybutyrate and cecal abundance of SCFA-producing taxa (25). Because butyrate is reduced in stool of patients, minocycline therapy could rectify SCFA production and thus reduce the severity of IC symptoms.

## Conclusions

In summary, UCPPS and IC/BPS are associated with dysbiosis of the gut and possibly other compartments. Future studies are needed to more definitively determine whether these associations are causally linked to pathogenesis. Mechanisms of visceral convergence that mediate organ crosstalk as well as circulating factors endow gut microbiota with the potential to modulate IC symptoms peripherally and centrally. By targeting the effects of dysbiosis directly and indirectly, microbiota thus offer unique therapeutic opportunities to potentially treat pelvic pain and comorbid conditions of IC.

## Version 1 09/02/2025

Electronic publication
